# A New Fibrin-Heparine Coated Self-Expanding Stent for the Rescue Treatment of Intracranial Stenosis—a Multicentric Study

**DOI:** 10.1007/s00062-024-01448-6

**Published:** 2024-08-23

**Authors:** Franziska Dorn, Yves Leonard Voss, Mousa Zidan, Stephanie Neuhaus, Nils Lehnen, Paul Stracke, Wolfram Schwindt, Mostafa Ergawy, Christian Dyzmann, Markus Moehlenbruch, Jessica Jesser, Dominik Vollherbst, Manuel Moreu, Carlos Pérez-García, Maxim Bester, Fabian Flottmann, Andreas Simgen, Stefan Schob, Ansgar Berlis, Christoph Maurer, Jan Hendrik Buhk, Hannah Hentschel, Christian Loehr, Bernd Eckert, Javier Saura, Fernando Delgado, Daniel Paech, Hannes Nordmeyer

**Affiliations:** 1 Department of Neuroradiology, University Hospital of Bonn, Bonn, Germany; 2 Department of Diagnostic and Interventional Neuroradiology, Städtisches Klinikum Solingen, Solingen, Germany; 3 Department of Interventional Neuroradiology, University Hospital Muenster, Muenster, Germany; 4 Neuroradiology Department, Sana Kliniken, Lübeck, Germany; 5 Department of Neuroradiology, Heidelberg University Hospital, Heidelberg, Germany; 6 Neurorradiología Intervencionista, Hospital Universitario Clínico San Carlos, Madrid, Spain; 7 Department of Diagnostic and Interventional Neuroradiology, University Medical Center Hamburg-Eppendorf, Hamburg, Germany; 8 Department of Diagnostic and Interventional Neuroradiology, Westpfalz-Klinikum, Kaiserslautern, Germany; 9 Department of Neuroradiology, University Hospital Halle, Halle (Saale), Germany; 10 Diagnostic and Interventional Neuroradiology, University Hospital Augsburg, Augsburg, Germany; 11 Department of Neuroradiology, Klinik St. Georg, Asklepios Hospital Group, Hamburg, Germany; 12 Department of Neuroradiology, Klinikum Vest, Recklinghausen, Germany; 13 Department of Neuroradiology, Klinik Altona, Asklepios Hospital Group, Hamburg, Germany; 14 Department of Radiology, HGU Gregorio Marañón, Madrid, Spain; 15 Neuroradiology Unit, Hospital Reina Sofía, Córdoba, Spain; 16 School of Medicine, Department of Health, Witten/Herdecke University, Witten, Germany

**Keywords:** ICAD, Rescue stent, Intracranial stenosis, Emergency sten ng, Surface modifica on

## Abstract

**Introduction:**

Rescue intracranial stenting is necessary to provide sufficient recanalization after mechanical thrombectomy (MT) in patients with acute large vessel occlusions (LVO) due to an underlying intracranial atherosclerotic disease (ICAD). The CREDO heal is a novel stent that provides a potentially lower thrombogenicity due to surface modification. We present the first multicentric experience with the CREDO heal for acute rescue stenting.

**Methods:**

Data of 81 patients who underwent rescue stenting after MT at 12 centers in Germany and Spain were prospectively collected and retrospectively evaluated.

**Results:**

Final mTICI 2b‑3 was reached in 95.1% after median two MT maneuvers and stenting. Four periprocedural complications resulted in clinical deterioration (4.9%). Intraparenchymal hemorrhage occurred in one patient (1.2%) and functional independence at FU was reached by 42% of the patients. Most interventions were performed under Gp IIb/IIIa inhibitors.

**Conclusion:**

CREDO heal was effective and safe in our case series. However, more data is needed to define the optimal antithrombotic regime. The use under single antiplatelet medication is not supported by our study.

## Introduction

Mechanical thrombectomy (MT) is the standard of care for selected patients with large vessel occlusions [1]. With technical advancements and new developments, the number of successful procedures is steadily increasing [2]. However, recanalization still fails in up to 20% of the cases and the prognosis of patients without sufficient reperfusion is poor [3, 4]. One reason why standard MT fails is an underlying intracranial atherosclerotic disease (ICAD) which accounts for 5 to 10% of all ischemic stroke cases in Europe and North America and up to 50% in the Asian population [5]. Since recanalization is one of the strongest predictors of good clinical outcome, rescue stent angioplasty is the only option to keep the vessel patent in this situation. Literature on rescue stenting is limited mainly due to retrospective series and a few prospective studies, in which a variety of different stent designs and techniques were used [6–8]. Surface modifications have recently been introduced to intracranial stents: the pEGASUS (Phenox, Wallaby, [9]), designed mostly for stent assisted coiling of cerebral aneurysms, and the CREDO heal (Acandis), designed to specifically target ICAD. The expectation for this technique is that stent implantation is less thrombogenic and therefore safer and potentially possible under single antiplatelet treatment (SAPT) thus reducing the risk for hemorrhagic complication in the acute phase.

We aim to proof the safety and efficacy of the CREDO heal in combination with the NeuroSpeed system in acute rescue stenting.

## Methods

### Patient Selection and Baseline Characteristics

Data of all patients who underwent rescue stenting with the CREDO heal between 08/21 and 06/22 at 12 tertiary stroke centers (10 in Germany, 2 in Spain) was prospectively acquired and retrospectively evaluated.

Inclusion criteria were:

1. presence of a residual hemodynamically relevant or re-occluding intracranial stenosis after MT
2. absence of intracranial hemorrhage
3. absence of contra-indications to treatment with antiplatelet agents

No other intracranial stents were used. The NeuroSpeed double lumen PTA balloon was used as the first line balloon in all cases and served as the delivery system for the CREDO heal stent. Both, anterior and posterior circulation lesions were included. When eligible, patients received weight adapted iv-tPA prior to the thrombectomy procedure. The administration of antiplatelet medication during and after the procedure was at the discretion of the treating physicians.

Baseline admission clinical parameters were assessed by the National Institute of Health Stroke Scale (NIHSS) and the modified Ranking Scale (mRS). All patients were examined by board certified neurologists and assessed according to the mRS. The extent of the ischemic lesion was assessed by the Alberta Stroke Program Early CT Score (ASPECTS) for anterior circulation strokes and by the posterior circulation Alberta Stroke Program Early CT Score (pc-ASPECTS) for posterior circulation strokes.

Ethics approval was obtained (University of Bonn, 394/22).

### Outcome and Procedural Parameters

The clinical outcome was assessed using the mRS at discharge and at follow up. Good clinical outcome was defined as mRS ≤ 2 at follow-up. In patients without imaging follow-up mRS values were acquired by telephone calls (mRS certified nurse). Documented angiographic parameters were: initial site of occlusion/stenosis, number of recanalization attempts before stenting, vessel diameter proximal and distal to the stenosis, grade of stenosis (according to WASID criteria) and any intracranial hemorrhage on postinterventional flat panel CT (FPCT). Successful recanalization was defined as mTICI ≥ 2b and rated by the respective centers. Time parameters in the aPTAS group were stroke onset to hospital admission, door to imaging, door to groin, door to recanalization, groin to final recanalization attempt and groin to end of procedure. Symptomatic intracranial hemorrhage was assessed according to the ECASS II criteria. Furthermore, mortality, procedural complications and device related complications (dissections, wire perforation, vessel rupture, and downstream territory embolisms) were evaluated.

### Interventional Procedure

All patients were treated under general anesthesia. Guiding catheters were 8F balloon guide and standard guide catheters. Aspiration or distal access catheters were used when chosen for thrombectomy or for navigational purposes in challenging anatomies. The degree of stenosis and the choice of NeuroSpeed PTA balloon diameter was made upon 2D angiographic measurements, in elective cases additionally on 3D Angiograms. The length of the stenosis was measured likewise and reassurance was obtained by microwire assisted measurement with help of an extracorporeal ruler.

### CREDO Heal Stent and NeuroSpeed PTA Balloon

The NeuroSpeed balloon is an over-the-wire double-lumen catheter with an inner diameter of 0.0165″ and a length of 150 cm. Balloon diameters range from 1.5 to 4.0 mm with a working length of 8 mm. The semi-compliance of the balloon allows for inflation to +/− 0.3 mm of the nominal size.

The CREDO heal stent is a self-expanding laser-cut nitinol stent available in sizes between 3.0 and 5.0 mm, passing through a 0.0165 inch lumen. Its radial force is adapted to the requirement of ICAD treatment and thus exceeding that of the regular Acclino Flex stent designed for aneurysm treatment and otherwise resembles the CREDO stent in terms of stent manufacturing.

The stent coating consists of a fibrin layer with covalently bounded heparin on the entire surface of the implant, which simulates the last step of natural hemostasis (mimikry) and thus aims at reducing local thrombogenesis as well as facilitating endothelialization.

## Results

### Baseline Characteristics

Eighty-one patients were included (34 female, 42%). 71.6% of all patients were between 60 and 89 years old, one patient was between the age of 18 and 29 years and 2 patients ages were between 30 and 39 years, 4 patients were older than 89 years. Ten patients (12.3%) were already on acetylsalicyc acid (ASA) 100 mg/d medication, 2 with Clopiogrel 75 mg/d (2.5%) and 2 with the combination of ASA 100 mg/d and Ticragelor 180 mg/d (2.5%) prior to the intervention. Seven patients were on anticoagulative medication prior to the intervention.

Baseline ASPECTS was 9 (minimum 5, maximum 10) and weight adapted iv-tPA was given to 19 of the patients before the intervention (23.5%).

Localization of the stent was the ACA in 2 (2.5%), MCA in 41 (50.6%, 23 M1-, 7 M2- and 1 M3-segment), PCA in 5 (6.2%, 2 P1-, 3 P2-segment), intracranial ICA in 12 patients (14.8%), BA in 15 (18.5%) and the V4-segment in 6 patients (7.4%).

Median NIHSS at admission was 15 (min. 1, max. 21).

Overall, 1–3 passes of any technique were performed before rescue stenting (median 2). Time from puncture to stent deployment was 82 ± 47 min. Table 1 summarizes the baseline characteristics.

### Antithrombotic Medication

**Fig. 1:**
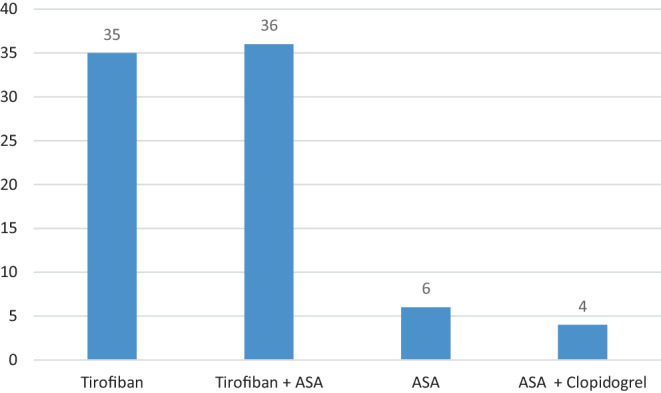
Periprocedural antithrombotic medication: Tirofiban in 35 patients, Tirofiban and ASA 500 mg in 36 patients, ASA 500 mg alone in 6 patients, ASA and Clopidogrel in 4 patients

Periprocedural antithrombotic medication included Tirofiban in 35 patients (43.2%) alone, Tirofiban and ASA 500 mg iv in 36 patients (44.4%), ASA 500 mg iv alone in 6 patients (7.4%), ASA and Clopidogrel in 4 patients (4.9%). Figure 1 summarizes the periprocedural antiplatelet management.

**Fig. 2:**
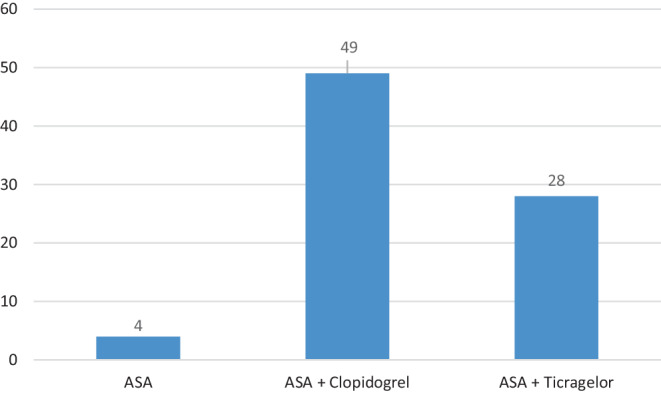
Postprocedural antithrombotic medication: ASA 100 mg/d in 4 patients, ASA 100 mg/d and Clopidogrel 75 mg/d in 49 patients, ASA 100 mg/d and Ticagrelor 180 mg/d in 28 patients

Postprocedural antithrombotic medication comprised single and dual antiplatelet therapy. Four patients (4.9%) received SAPT with ASA 100 mg/d, all other patients received DAPT (ASA 100 mg/d and Clopidogrel 75 mg/d) in 49 patients (60.0%) and ASA 100 mg/d and Ticagrelor 180 mg/d in 28 patients (34.6%). Responder status was tested in 26 of the patients who were treated with ASA and Clopidogrel. Figure 2 summarizes the postprocedural antiplatelet management.

### Complications

Device-related technical problems occurred in 3 patients (3.7%); in 2 patients, the stent did not open completely (2.4%), which was solved by overlapping implantation of another stent (final mTICI 3 in both). In one patient (1.2%) the transport wire was hooked onto the stent struts which resulted in stent dislocation and required overlapping implantation of another stent. Finally, both stents got thrombosed (mTICI 0).

There was one extracranial ICA dissection that was treated with a stent.

One patient developed progressive fatal ICH and minor SAH was documented in 4 cases. In-stent thrombus formation was documented in 5 patients (6.1%); in 3 of these five patients no antiplatelet medication was given prior to but only immediately after stent placement and ASA iv in two of them; all but one thrombus dissolved after IV Gp IIb/IIIa inhibitors were administered. Dissection of the target vessel was documented in 2 patients (2.5%), both with a final mTICI 2c or 3 result. Vessel perforation requiring permanent or transient coil occlusion was documented in 1 patient (1.2%).

In total, periprocedural complications occurred in 11 patients (13.5%) in total:

- device related (3; 2 without clinical symptoms)
- extracranial ICA dissection (1; without clinical symptoms),
- thrombus in stent (5; 4 resolved under Gp IIb/IIIa antagonists),
- vessel perforation [1]
- fatal ICH [1]

In total, 4 of the periprocedural complications (4.9%) were associated with a clinical decline.

### Angiographical and Clinical Results:

Final recanalization result was mTICI 0 in three patients (3.7%), mTICI 2a in one (1.2%), mTICI 2b in 11 (13.6%), 2c in 11 (13.6%) and mTICI 3 in 55 patients (67.9%). Overall, mTICI 2b‑3 was reached in 77 patients (95.1%).

Stenosis grade prior to rescue stenting was 89 ± 9.7% and improvement of the stenosis was 72.8 ± 26%. Median vessel diameter was proximal 2.6 ± 0.83 and 2.2 ± 0.81 mm distal from the stenosis.

NIHSS at discharge was available in 65 patients (median 5.7, min. 0, max. 17). Ten patients died during the initial hospital stay. mRS at admission was available in 68 (84.0%) of the patients and follow up mRS after 30–280 days was available in 67 (82.7%) of the patients. Overall, 34 patients (42.0%) reached functional independence (mRS 0–2).

## Discussion

Prospective randomized trials showed superiority of aggressive medical management over intracranial angioplasty and stenting in patients with symptomatic ICAD [10, 11]. The superiority of the conservative treatment persisted over a period of at least 4 years [12]. However, subsequent prospective registry data proofed much lower complication rates and more favorable clinical outcomes for stent angioplasty if ICAD patients were selected carefully [13, 14].

Factors such as patient referral to high volume centers with higher operator experience, a longer time between the qualifying clinical event and the procedures of at least 7 days, under sizing the angioplasty balloon in order to avoid perforator occlusions (“snow plowing effect”) and the prevention of exchange maneuvers with the potential risk of distal wire perforations could reduce the risk for periprocedural complications and thus improve the clinical outcome. For the time being, there is reluctance to stent patients with symptomatic ICAD and current guidelines only recommend it for patients who are symptomatic even under best medical treatment [15]. However, in the case of intracranial stenosis leading to an acute symptomatic vessel occlusion, stent angioplasty is the only way to achieve effective and durable recanalization and thereby giving the patient a chance for a good clinical outcome. Stracke et al. published a series of 210 rescue stenting patients from seven international centers with a successful recanalization rate (at least TICI 2b) in 82.9% and a good clinical outcome in 44.8% of the patients [7]. In the recently published matched-pair SAINT analysis [16], favorable outcomes of patients who were treated by means of rescue intracranial stenting was 5‑fold higher (34.6%) with rescue stenting when compared to non-recanalized patients (6.5%, *p* < 0.001). The multicentric prospective ReSet registry (Rescue Stenting for failed endovascular thrombectomy) included 78 patients who required intracranial stenting after thrombectomy; the technical success was very high (mTICI 2b/3 in 98.7%) and a favorable outcome was reached in 66.7% and thereby almost twofold of the SAINT registry [8]. Preham et al. summarized four comparative studies with 352 patients in total and also found significantly higher rates for favorable outcome and lower mortality in the rescue stenting group [17]. In our study, recanalization was successful in 95.1% and 42% of the patients had a favorable outcome (mRS 0–2), both inferior to the ReSet results. In ReSet, 82% of the stents were patent on follow up and stent patency was an independent factor for a favorable outcome (OR 87.6; 95% CI 4.77 to 1608.9; *p* = 0.003). In our cohort, stent thrombosis occurred in four of the patients (4.9%); in three of these patients the stent was implanted before any antiplatelet medication was given and thrombosis resolved rapidly with Gp IIb/IIIa inhibitors. Interestingly, in ReSet, stent patency was significantly associated with periprocedural IV Gp IIb/IIIa inhibitors (OR 5.72; 95% CI 1.45 to 22.6; *p* = 0.013), which is in line with our results: the vast majority of the procedures (87.7%) were performed under Gp IIb/IIIa inhibitors (either with or without additional ASA medication) and in these cases all of the stents stayed patent.

Hemorrhagic complications were more frequent in series where patients were treated with rescue stenting (10.1% in the series by Stracke et al., 12.1% in the meta-analysis by Premat et al.) when compared to the randomized trials on mechanical thrombectomy that were summarized in the HERMES registry (0–8%). On the other hand, real life registries on MT, e.g. from the German Stroke Registry and from ETIS, in general report higher hemorrhagic rates [18, 19] when compared to the randomized trials.

It may sound contradictory to treat patients with an acute stroke and a subsequent high risk of hemorrhage with highly potent antiplatelet medication.

However, in a series of 27 patients, the risk for hemorrhage after rescue stenting was almost four times higher when Gp IIb/IIIa antagonists were *not* given when compared with patients who underwent the intervention under Gp IIb/IIIa antagonists [20]. Also, in the meta-analysis by Preham et al. the risk for intracranial hemorrhage was not increased [17]. In a systematic review on rescue stenting it was found that Gp IIb/IIIa antagonists were given in 89% of the patients and 95% of the patients received antiplatelet therapy after the procedure [21]. The rate of hemorrhage was very low in our series: only one patient developed massive and fatal ICH (1.2%). Minor SAH which did not require further intervention was documented in 4 cases (4.9%). Furthermore, our data supports the conclusion, that sufficient antiplatelet therapy ensures stent patency, thus limiting the infarct volume and subsequently reducing the risk of hemorrhage. A sub analysis from the ETIS registry showed that patients chances for a good outcome were the highest when rescue stenting of the basilar artery was performed after the 1st pass and decreased significantly after 2 or more passes [22]. In our cohort, median passes before rescue stenting were 2 (1–3) with a median time from groin puncture to recanalization of 82+/− 47 min. The fact that no more than 3 passes were performed in any procedure before the stent decision was made speaks for the high expertise of the participating centers on the one hand and is a possible explanation for the good clinical results compared to other series on the other.

Of course, the use of intracranial stents under single antiplatelet medication (SAPT) would be desirable in order to further reduce any hemorrhagic complication. The HEAL Technology mimics the final step of natural hemostasis and thus the conversion of fibrinogen to fibrin [23]. The controlled growth process results in a thin and fully polymerized network of fibrin with additional covalently bound heparin molecules [24]. This composition of the HEAL Coating results in the antithrombogenic and endothelialisation-promoting properties that are essential for the rapid healing of an intracranial device. Fibrin passivates the surface so that the implant is not perceived as a foreign body and neither inflammatory nor coagulative processes are triggered. Passivation thus leads to a significant reduction in thrombogenicity [25]. At the same time, the fibrin network forms an ideal scaffold for surrounding endothelial cells and thus supports endothelialisation and healing of the implant in the vessel. The covalently bound heparin reduces platelet activation and activation of the coagulation cascade. Thus, heparin also contributes to the antithrombogenic property of the HEAL Coating and thus of the device. The HEAL Coating or components of the coating are not released into the system after implantation of the device. Thus, the coating is not eluted and has no pharmacological effect [26].

The HEAL coating (and other coating technologies) were first developed for flow diverters with the idea to reduce the thrombogenicity, enhance endothelialization and potentially allow the use under SAPT or early dose reduction of DAPT if needed. Because of the need for DAPT, flow diversion is currently not indicated for the treatment of acutely ruptured aneurysms. Several case series showed that SAPT is reasonably safe in surface modified flow diverters [27] and a meta-analysis of 59 patients who were treated with different flow diverters with different surface technologies did not show any differences between single antiplatelet therapy (SAPT) and DAPT regimens with respect to periprocedural thromboembolic complications [28]. Randomized data however is not available, but the COATING study is currently enrolling patients with the intention to compare safety and efficacy of coated flow diverters under SAPT with bare flow diverters under DAPT [29].

So far, only one case has been published where a DERIVO flow diverter with HEAL technology was implanted under SAPT and no thromboembolic event was observed [26].

One of the biggest unanswered questions so far in this context is which antiplatelet medication is the safest and most effective to prevent thrombotic complications. A recently published meta-analysis included 237 patients where flow diverter stents with surface modifications were implanted under single antiplatelet medication and showed very low hemorrhagic complications (0.1%) and thromboembolic complications in 7.6%; thromboembolic events rate was much lower under Prasugrel (2.4%) and Ticagrelor (4.2%) when compared to ASA (20.2%). The authors conclude that SAPT in patients undergoing FD treatment for cerebral aneurysms has an acceptable safety profile, especially with the use of ADP-receptor antagonists [30].

Another open question is whether or not we can transfer the results on SAPT in flow diverters one-to-one to intracranial stents for the treatment of ICADs, as the underlying pathology is completely different. In our study, the vast majority of the patients were treated under Gp IIb/IIIa antagonists and in all of the cases the stents were patent. Postprocedural, almost all patients received DAPT (either ASA and Clopidogrel or ASA and Ticagrelor); we did not see any immediate thrombotic complication in these patients as well and on follow up (FU) all stented vessels were patent; however, FU was not available for 17.3% of the patients, which is why we are very cautious in interpreting these results.

## Conclusion

CREDO heal is the first intracranial stent for the treatment of ICAD with surface modification and was designed with the intention to offer lower thrombogenicity and enhance endothelialization. Our multicentric study proofs safety and efficacy of the novel CREDO heal stent system for rescue stenting of patients with LVOs and an underlying ICAD. The study showed that (most likely due the highly thrombogenic nature of the underlying lesions) sufficient antiplatelet medication is still mandatory despite the potential benefits of crosslinked fibrin coating. In keeping with existing evidence from the literature, we saw that the use of Gp IIb/IIIa antagonist had the highest impact on outcome and stent patency. Further studies are necessary to evaluate the safety and potential advantages of using the device under SAPT.

**Table 1:** Summary of the baseline data

		*n* = 81
Sex	Female	34 (42.0%)
	Male	47 (58.0%)
Age	18–29	1
	30–39	2
	40–49	4
	50–59	12
	60–69	12
	70–79	21
	80–89	25
	< 89	4
Localization	ACA	2 (2.5%)
	MCA	41 (50.6%)
	PCA	5 (6.2%)
	Intracranial ICA	12 (14.8%)
	BA	15 (18.5%)
Final TICI	0	3 (3.7%)
	1	0
	2a	1 (1.2%)
	2b	11 (13.6%)
	2c	11 (13.6%)
	3	55 (67.9%)
Rt-PA prior to the intervention	Yes	19 (23.5%)
	No	65 (76.5%)
